# The Bacterial Preparation OK432 Induces IL-12p70 Secretion in Human Dendritic Cells in a TLR3 Dependent Manner

**DOI:** 10.1371/journal.pone.0031217

**Published:** 2012-02-21

**Authors:** Arnt-Ove Hovden, Marie Karlsen, Roland Jonsson, Silke Appel

**Affiliations:** Broegelmann Research Laboratory, The Gade Institute, University of Bergen, Bergen, Norway; Carl-Gustav Carus Technical University-Dresden, Germany

## Abstract

Dendritic cells (DC) used in therapeutic cancer immunotherapy have to be able to stimulate T cells resulting in an immune response that can efficiently target the cancer cells. One of the critical hurdles has been the lack of IL-12p70 production when maturating the DC, which is rectified by using the bacterial preparation OK432 (trade name Picibanil) to mature the cells. In order to identify the mechanism behind OK432 stimulation of DC, we investigated the contribution of different TLR to examine their involvement in IL-12p70 production. By combining different inhibitors of TLR signaling, we demonstrate here that TLR3 is responsible for the IL-12p70 production of DC induced by OK432. Moreover, our data suggest that the ligand triggering IL-12p70 secretion upon TLR3 stimulation is sensitive to proteinase and partly also RNAse treatment. The fact that a bacterial compound like OK432 can activate the TLR3 pathway in human DC is a novel finding. OK432 demonstrates a critical ability to induce IL-12p70 production, which is of great relevance in DC based cancer immunotherapy.

## Introduction

Dendritic cells (DC) are the sentinels of the immune system and at the crossroad of the innate and adaptive immunity. Due to their outstanding capacity to stimulate T cells, there is a considerable interest of employing these qualities in various forms of immunotherapy [1], [2]. In DC-based cancer immunotherapy one of the critical hurdles has been the lack of IL-12p70 production when stimulating the DC with the Jonuleit cytokine cocktail (IL-1β, IL-6, TNF-α and PGE_2_ [3], which is the most commonly used maturation stimulus in clinical trials. To find a better way to stimulate DC used in cancer immunotherapy, a range of stimuli has been tested [4]. The maturation stimulus of choice must induce a functional maturity of the DC resulting in a superior T cell stimulation that can efficiently target the cancer cells. To fulfill these criteria we have investigated the low-virulence strain of penicillin-killed *Streptococcus pyogenes* (OK432) [5]. OK432 is available as a licensed drug (trade name, Picibanil) and has been used efficiently to treat a variety of tumors [6], [7] both alone or in combination with chemotherapy [8]. The effect of OK432 in cancer patients has not been thoroughly investigated, but we have recently shown that OK432 induces production of substantial amounts of IL-12p70 and other inflammatory cytokines by human monocyte-derived DC *in vitro* [9]. Furthermore, OK432 induces up-regulation of surface expression of MHC class II molecules as well as a number of co-stimulatory molecules on DC. Moreover, OK432 stimulated DC have an increased T cell stimulatory capacity [9].

The mechanism behind OK432 stimulation of DC at the cellular level is not well understood, and there is conflicting evidence in the literature. Both p38 MAPK, TLR4 and β_2_ integrin have been proposed as the key component in OK432 mediated DC maturation and cytokine release [10]–[14], possibly in an orchestrated manner as β_2_ integrin has been shown to co-localize with TLR4 in lipid rafts in mouse β hepatocytes [15]. However, an antitumor effect of OK432 was also seen in TLR4−/− mice [14] and Nakahara and co-workers found OK432 to act independent of TLR2 and TLR4, but dependent on β_2_ integrin [11]. The active ingredient of OK432 has been proposed to be a lipoteichoic acid-related molecule [16] which would normally point to an involvement of TLR2 [17].

To ease the transition of OK432 into a clinical protocol using OK432 stimulated DC in therapeutic cancer vaccination, the aim of this study was to explore the mechanisms behind the ability of OK432 to stimulate DC and to elicit high IL-12p70 secretion. To this end we investigated the contribution of different TLR in OK432 mediated IL-12p70 production. By combining inhibitors of TLR signaling, we were able to demonstrate that TLR3 is responsible for the OK432 induced IL-12p70 production by DC. We have also obtained evidence that the ligand triggering IL-12p70 secretion upon TLR3 stimulation is sensitive to proteinase and partly also RNAse treatment.

## Results

### Chloroquine, but not TLR4 and MyD88 inhibition, abrogates OK432 mediated IL-12p70 production

In order to decipher the mechanism behind OK432 stimulation of DC, we utilized various compounds inhibiting TLR signaling, as TLR involvement was likely with a bacterial compound like OK432. As the induction of IL-12p70 was of high interest, we chose IL-12p70 secretion as the primary readout, and expression of cell surface markers as secondary readout only.

**Figure 1:**
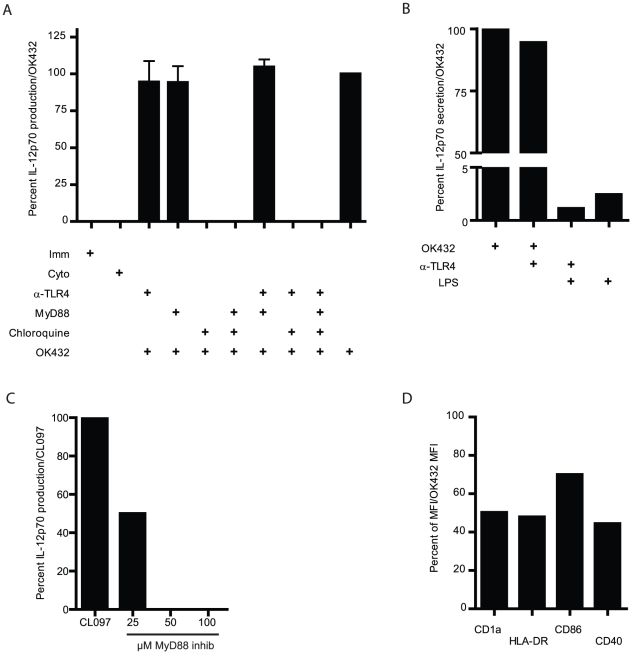
Chloroquine treatment abolishes IL-12p70 production by DC stimulated with OK432. A) Combinations of blocking antibody to TLR4, MyD88 inhibitory peptide and chloroquine were used to decipher contributions of different TLR in IL-12p70 production by DC after OK432 stimulation. The DC were pre-treated with the indicated compounds for 30 minutes at 37°C before maturation was induced by OK432. Immature DC were given only IL-4 and GM-CSF and Cyto were stimulated with the Jonuleit cytokine cocktail (IL-1β, IL-6, TNF-α and PGE_2_). Only treatment with chloroquine reduced the level of IL-12p70 produced after OK432 stimulation. Mean+SEM is shown for 4 donors. B) To verify the effect of TLR4 blockade, a blocking antibody against TLR4 was used at 9 µg/ml. This reduced the level of LPS (20 ng/ml) induced IL-12p70 production by 50%. Mean of n = 2. C) MyD88 inhibition effectively decreases CL097 mediated IL-12p70 production via TLR7/8 signaling. D) Chloroquine has profound effects on the maturation status of OK432 stimulated DC. Percent MFI of CD1a, HLA-DR, CD86 and CD40 after pre-treating the DC with chloroquine before OK432 stimulation compared to OK432 alone (set to 100 percent) is shown. One representative experiment shown of three performed.

To verify or exclude involvement of TLR4, we utilized a blocking antibody against this receptor. However, blocking of TLR4 failed to reduce the level of IL-12p70 production after OK432 stimulation (Figure 1A). In order to ensure that the antibody was functional and the concentration used was sufficient, we included LPS as a positive control. Incubation of the cells with the blocking antibody against TLR4 reduced the level of LPS induced IL-12p70 secretion by 50% (Figure 1B).

Most TLR signal via the MyD88 adaptor protein, with the exception of TLR3 and one of the pathways of TLR4 [18]. In order to block all signaling via MyD88, an inhibitory peptide was utilized. The MyD88 inhibitory peptide was toxic at high concentrations and therefore titrated to not influence cellular viability, with an optimal concentration found to be 100 µM. At this concentration the inhibitory MyD88 peptide did not reduce the level of IL-12p70 detected after OK432 stimulation (Figure 1A), demonstrating that OK432 induced IL-12p70 production is not mediated via the MyD88 pathway. To ensure that the concentration used was sufficient to block signaling via MyD88, we used MyD88 dependent TLR7/8 ligand CL097 as positive control. The inhibitory peptide completely abolished IL-12p70 production induced by CL097 (Figure 1C).

Chloroquine is a compound that recently has been shown to stop signaling of TLR3 and 9 localized in endosomes by directly binding of nucleic acids masking their TLR-binding epitope [19]. Due to its toxicity the chloroquine was titrated to achieve efficient signaling blockade of the endosomes, while not influencing the cellular viability. The optimal concentration was found to be 40 µM both for immature and stimulated DC. At this concentration, chloroquine completely blocked all IL-12p70 production after OK432 stimulation, demonstrating that the production of IL-12p70 is dependent on a functional endosome (Figure 1A). Inhibition with chloroquine also led to a reduction of median fluorescence intensity (MFI) of surface CD40, CD1a, and HLA-DR by around 50% and a reduction of CD86 MFI by 30% (Figure 1D). Little effects were seen on CD83, CD80, CCR7 and CD38 expression (data not shown). This, together with the IL-12p70 reduction, demonstrates a phenotypic effect and a reduced maturation of OK432 stimulated DC pre-treated with chloroquine.

### TLR3 siRNA inhibit the IL-12p70 production after OK432 stimulation in a dose-dependent manner

**Figure 2:**
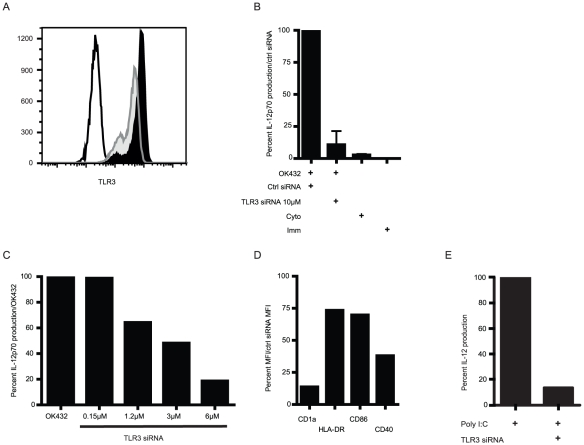
TLR3 knockdown has a considerable effect on OK432 induced IL-12p70 production by DC. A) DC were electroporated with either control siRNA that has no specific target in human cells or with two siRNA specific against TLR3. The level of siRNA mediated TLR3 knockdown was found to be around 40 percent after 24 hours of OK432 stimulation (48 h after electroporation). Open histogram: unstained control; shaded histogram: mock treated; black histogram: TLR3 siRNA treated; one representative experiment shown of five. B) After resting for 24 hours, the DC were incubated with OK432 for 24 hours and the supernatant was collected and analyzed for IL-12p70 production by ELISA. OK432 is critically dependent on TLR3 signaling to induce IL-12p70 production. Immature cells (Imm) received only IL-4 and GM-CSF, the Cyto group were matured with IL-1β, IL-6, TNF-α and PGE_2_. Mean+SEM are shown for four donors. C) To further verify the specificity of the TLR3 siRNA knock down, DC were electroporated with escalating concentrations of TLR3 siRNA and rested for 24 hours. Subsequently, after 24 hour stimulation with OK432, IL-12p70 production was analyzed. One representative donor is shown out of three. D) TLR3 siRNA knockdown has a marked effect on the phenotype of OK432 matured DC. Percent MFI of CD1a, HLA-DR, CD86 and CD40 after TLR3 siRNA knock down and OK432 stimulation compared to OK432 plus control siRNA (set to 100%). Mean of two donors shown. E) TLR3 siRNA inhibit IL-12p70 production upon polyI∶C stimulation of DC. IL-12p70 production of polyI∶C treated DC were set to 100%. One representative donor is shown.

The previous experiments indicated involvement of either TLR3 or MyD88 independent TLR4. To resolve this, siRNA against TLR3 was utilized to block signaling via TLR3. Two different TLR3 siRNA were electroporated 24 hours prior to OK432 stimulation. The level of protein expression of TLR3 was reduced by around 40% after 48 h (harvest of cells and medium) as measured by flow cytometry (Figure 2A). The TLR3 siRNA were able to reduce the level of IL-12p70 production by 80–100% after OK432 stimulation (Figure 2B). This effect was specific as a control siRNA did not reduce IL-12p70 levels, and the effect was dose dependent (Figure 2C). The knock down effect was also obvious on a number of surface markers associated with DC maturation with reduction of expression from 25 to over 80% (Figure 2D). As a control of the successful TLR3 knock down, 50 µg/ml polyI∶C was used as a positive control to stimulate TLR3 siRNA treated DC. The level of IL-12p70 was reduced by close to 90% (Figure 2E). Moreover, the effect was not due to differences in viability, as the siRNA treated cells produced the same amounts of IL-8 as mock treated DC (Figure S1).

### The TRIF pathway is critical to elicit IL-12p70 production

**Figure 3:**
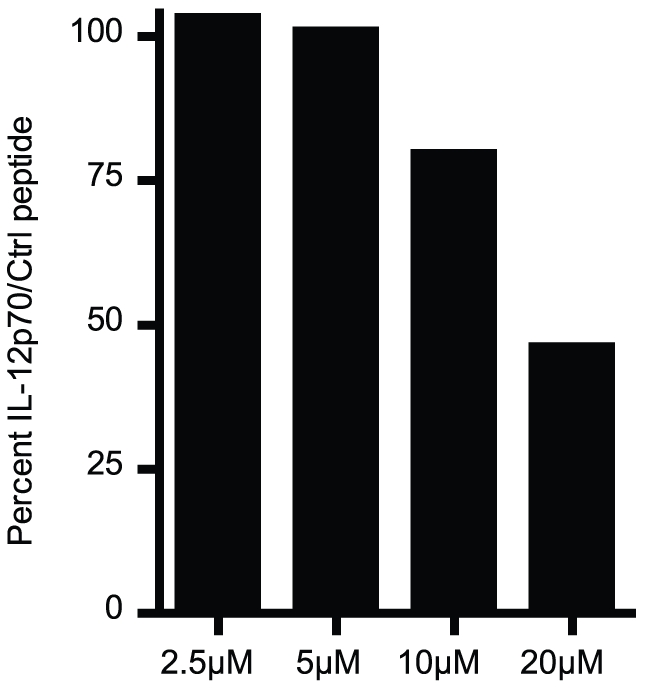
OK432 induced IL-12p70 production is TRIF dependent. Pre-treating the DC for 30 minutes at 37°C with an inhibitory peptide of TRIF signaling reduced the level of OK432 induced IL-12p70 production in a dose dependent manner compared to a control peptide and OK432. Concentrations higher than 20 µM had a toxic effect on the DC. Data from one representative donor is shown.

To further strengthen the involvement of TLR3 signaling in OK432 stimulation of DC, a TRIF inhibitory peptide was employed to demonstrate dependence on this pathway for IL-12p70 secretion. The TRIF inhibitory peptide and the control peptide were carefully titrated as they both were toxic to the DC at high concentrations. At an optimal concentration, the TRIF inhibitory peptide showed an inhibition of the IL-12p70 production upon OK432 stimulation in a dose dependent manner, further demonstrating that OK432 mediated IL-12p70 production is dependent on a functional TRIF pathway (Figure 3). Only little effect was seen on the phenotype of the cells (data not shown).

### OK432 mediated IL-12p70 production is dependent on intact RNA and protein present

**Figure 4:**
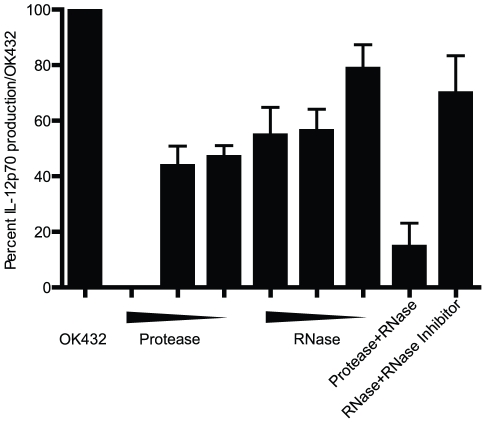
OK432 mediates its effect via TLR3 by a ligand that needs RNA and protein present. OK432 was pre-treated with RNase A and protease K for 30 minutes at 37°C before being diluted 25-fold and added to the DC. The pre-treatment was performed with 100, 10 and 2 µg/ml protease K, and RNase A was tested at 100, 10 and 1 µg/ml. After 24 hours the supernatants were analyzed for presence of IL-12p70. The amount produced is displayed as a percentage compared to that of OK432 alone set to 100%. Protease K treatment diminished IL-12p70 production in a dose-dependent manner. RNase reduced the IL-12p70 production by more than 50%, while the combination of protease K and RNase treatment reduced the IL-12p70 production by around 90%. IL-12p70 production was rescued by adding a RNase inhibitor together with the pre-treatment. For each treatment, the mean and SEM is shown (N = 2–4 experiments).

To elucidate the nature of the TLR3 ligand by which OK432 stimulates DC to produce IL-12p70, the OK432 compound was treated with combinations of protease K, DNase I and RNase A prior to addition to DC. With OK432 alone set to 100%, DNase treatment of OK432 had no effect on IL-12p70 secretion (data not shown). Protease K reduced IL-12p70 production by 50–100% in a dose dependent manner, whereas RNase A reduced IL-12p70 production by 20–50% depending on the concentration (Figure 4). In combination, protease and RNase A reduced IL-12p70 secretion by almost 90%. The effect of RNase A treatment of OK432 on IL-12p70 production could be rescued by adding an RNase inhibitor into the pre-treatment mix. Therefore, our data suggest that the bacterial compound OK432 exerts its effect on TLR3 mediated IL-12p70 production with a ligand that requires protein and is augmented by the presence of intact RNA.

## Discussion

OK432 has been used clinically for several decades against malignant and benign neoplasms with excellent safety profile [5]–[8], [10], [20]. In the last decade, it has also attracted some interest as maturation stimulus in DC-based therapeutic cancer vaccination protocols [9], [11], [14], [21]–[24]. However, until now the mechanism behind OK432 stimulation of DC has been elusive.

We therefore set out to work out the pathways activated by OK432. Our inhibition experiments demonstrated that OK432 and IL-12p70 production is dependent on a functioning endosome. The MyD88 inhibition showed that IL-12p70 production is not dependent on MyD88 signaling and therefore point to TRIF dependent TLR4 signaling or TLR3. However, if OK432 had been dependent on TLR4, the IL-12p70 should not be completely blocked by chloroquine as demonstrated. CCL5/Rantes has been linked to TLR3 activation in epithelial cells [25] and consistently, we have previously found a marked up-regulation of CCL5 upon OK432 stimulation of DC [9]. The fact that a significant antitumor activity after OK432 treatment was also seen in TLR4−/− mice [14] points to other TLR having a significant role in the induction of an immune response. Furthermore, the level of IL-12p70 induced by LPS via the TLR4 pathway was only about 3% of that of OK432 alone. We observed an almost total block of IL-12p70 secretion after TLR3 knock down. This effect was specific as a control siRNA did not reduce IL-12p70, and the effect was dose-dependent. TRIF inhibition was also shown to be dose-dependent, but due to the cellular toxicity of the inhibitory peptide, only a 50% reduction of IL-12p70 production was achieved. Comparison of OK432 and TLR3 ligand polyI∶C revealed that polyI∶C was inferior to OK432 in eliciting IL-12p70 production (data not shown), consequently, exchanging OK432 with polyI∶C in a maturation cocktail would not be sufficient to induce equivalent amounts of IL-12p70.

OK432 mediated maturation of DC has been attributed to several receptors, like TLR4, p38 MAPK and the β_2_ integrin [10]–[14]. The active component of OK432 has been suggested to be a lipoteichoic acid-related molecule [16], normally a ligand for TLR2 [17]. Nakahara and co-workers found OK432 stimulation to be independent of TLR2 and TLR4 [11], which is in line with our finding that TLR3 is a critical part of OK432 induced IL-12p70 production. As we are interested in the use of OK432 as part of a clinical protocol, we chose to focus on the IL-12p70 production as it is critical to ensure a good cytotoxic T cell response [26]. This does not exclude other pattern recognition receptors (PRR) to recognize components of OK432 that contribute to DC maturation. As OK432 is a compound made from a whole bacterium it is to be expected that several PRR participate in subsequent DC signaling. This is substantiated by the fact that TLR3 siRNA knock down, chloroquine treatment and TRIF inhibition sufficient to reduce IL-12p70 production by 50–100% only to some extent showed an effect on the DC phenotype.

Other PRR that recognize nucleic acids like RIG-I-like RNA helicases and melanoma differentiation-associated gene 5, reside in the cytosol [27], [28] and therefore no effect should be seen after chloroquine, TLR3 siRNA and inhibitory TRIF treatment. That does not exclude other PRR like nucleotide-binding oligomerization domain containing 2 (NOD2) augmenting IL-12p70 production, like Tada *et al.* reported for NOD2 ligands and TLR including TLR3 [29]. It is also possible and even likely that other PRR contribute to the induction of the inflammatory environment seen after OK432 stimulation of DC. Although TLR3 induced IRF3 has been verified as an important mechanism to induce type I interferons such as IFN-β [30], [31], also NOD2 has been found to induce IRF3 [32]. Moreover, TLR3 induced NF-κB and AP-1 is responsible for induction of pro-inflammatory cytokines [33].

The ligand for TLR3 is normally considered to be viral dsRNA over 40–50 nucleotides long, due to the distance between dimers of TLR3 [34], [35]. OK432 could harbor RNA in a manner untypical of a bacterium, either intrinsically, or as a consequence of the OK432 manufacturing process. Our data suggest that the ligand from OK432 mediating IL-12p70 production via TLR3 is sensitive to RNase A, which has ssRNA specificity under physiological conditions [36] and protease K. As both protein and RNA need to be present, one may speculate that a bacterial protein is needed to promote the correct secondary structure of bacterial RNA in order to have an efficient TLR3 ligation. This is supported by the fact that also mRNA has been reported to be able to activate TLR3 mediated signaling [37] and Marshall-Clarke co-workers reported that in murine immune cells, including DC, the single stranded synthetic polyinosinic acid could mediate signaling via TLR3 [38].This is also in concordance with our observation that reconstituted OK432 loses its IL-12p70 eliciting capacity rapidly over days stored at 4°C. Furthermore, Derbigny and co-workers have recently reported TRIF dependent IFN-β production after *Chlamydia* infection of murine macrophages and attributed this to TLR3 mediated signaling [39]. It has also been suggested that dsRNA from helminths can activate TLR3 in murine DC [40].

In conclusion, our results together with the above mentioned study by Derbigny *et al.* suggest that TLR3 signaling is a common feature for both murine and human immune cells in response to at least some bacteria. This can have direct consequences for the ongoing quest to find suitable maturation stimuli for DC-based therapeutic cancer vaccines. OK432 is certainly able to induce a range of inflammatory mediators, among them the critical IL-12p70, a key cytokine in eliciting cytotoxic T cell mediated immunity.

## Materials and Methods

### DC generation

DC were generated from monocytes isolated from buffy coat preparations from healthy blood donors (Blood Bank, Haukeland University Hospital, Bergen, Norway) as described [9]. Briefly, peripheral blood mononuclear cells were separated by a density gradient centrifugation and the monocytes were then negatively isolated using the Dynabeads Untouched Human Monocytes (Invitrogen, Carlsbad, CA) following the manufacturer's instructions. The monocytes were cultured with IL-4 (20 ng/ml; Immunotools, Friesoythe; Germany) and GM-CSF (100 ng/ml; Immunotools, Friesoythe; Germany) in RP10 medium [RPMI 1640 (Cambrex Bioscience, Verviers, Belgium) with 10% FCS (PAA, Pasching, Austria); 100 units/ml penicillin and 100 µg/ml streptomycin (Sigma-Aldrich, St. Louis, MO)] for 5–6 days to generate immature DC. Cytokines were replenished every 2–3 days. The maturation of the cells was performed for 24 hours with the Jonuleit cytokine cocktail [IL-1β, 10 ng/ml; IL-6, 1000 U/ml; TNF-α 10 ng/ml (all from Immunotools, Friesoythe; Germany) and PGE_2_, 1 µg/ml (Sigma-Aldrich, St. Louis, MO)] or with OK432 (Picibanil, Chugai Pharmaceutical Co. Ltd, Tokyo, Japan). The dosage of OK432 was 0.1 Klinische Einheit (KE), where 1 KE equals approximately 0.1 mg.

### Inhibition of TLR signaling

To block TLR4 signaling, a polyclonal blocking antibody (Anti-hTLR4-lgA, Invivogen, San Diego, CA) was used at a titrated concentration, 9 µg/ml. Chloroquine (Sigma-Aldrich, St. Louis, MO) was utilized to inhibit TLR signaling from the endosomal compartment at a titrated, non-toxic concentration (40 µM). To analyze any involvement of signaling via MyD88, a blocking peptide hindering MyD88 dimerization was utilized (100 µM, IMG-2005, Imgenex, San Diego, CA). A TRIF inhibitory peptide (Pepinh-TRIF, Invivogen) was used to block signaling via the TRIF pathway at a titrated concentration of 50 µM. Appropriate control peptides were included in all experiments. Two TLR3 siRNA (Invitrogen, Carlsbad, CA) were used at 5 µM each, delivered by square wave electroporation (500 V, 1 ms; ECM 830, BTX, Holliston, MA). TLR3 down-regulation was analyzed after 24 and 48 hours by intracellular staining of TLR3. OK432 and cytokine cocktail matured DC were included in all experiments as controls and the level of IL-12p70 production was normalized to production of OK432 treated DC to compensate for donor variations. In order to disclose the nature of the ligand responsible for inducing IL-12p70 production, OK432 was pre-incubated with DNase I (Sigma-Aldrich, St. Louis, MO), RNase A, RiboLock nuclease inhibitor (2 U/µl) and protease K (all Fermentas, St. Leon-Rot, Germany). The concentrations used were 100 µg/ml, 10 µg/ml and 2 µg/ml of protease in 40 µl volume; 100 µg/ml, 10 µg/ml and 1 µg/ml RNase A in 40 µl and 2 U DNase in 10 µl for 30 minutes at 37°C. This was followed by diluting OK432 to a final concentration of 0.1 KE in 1 ml total volume. To avoid unwanted degradation of OK432 no inactivating of enzymes was performed.

### Flowcytometry

Immunostaining was performed as described previously [9]. Briefly, the cells were incubated with titrated amount of antibodies for 10 minutes at room temperature before the cells were washed and immediately analyzed on a FACSCanto I cytometer (BD Biosciences, Heidelberg, Germany). All subsequent analyses were done with FlowJo software (Tree Star, Ashland, OR). The antibodies used were CD1-PE (NA1/34-HLK), CD14-FITC (UCHM1), CD38-Alexa Fluor 647(AT13/5), CD86-FITC (BU63), CD83-PE (HB15e), CD80-APC (MEM-233), CD40-FITC (LOB7/6), HLA-DR-APC (HL-39; all from AbD Serotec, Düsseldorf, Germany), and CCR7-PE (150503; R&D Systems, Minneapolis, MN).

### Cytokine determination

A sandwich ELISA was used to determine the amount of secreted IL-12p70 (BioLegend, San Diego, CA) according to the manufacturer's instructions.

## Supporting Information

Figure S1 **TLR3 siRNA treated DC produce similar amounts of IL-8 as mock treated DC.** DC were either mock treated or electroporated with either control siRNA that has no specific target in human cells or with two siRNA specific against TLR3. IL-8 production was measured by commercially available sandwich ELISA (R&D systems) to evaluate the viability of the cells. The amount IL-8 produced is displayed as a percentage compared to that of mock treated cells set to 100%. Mean and SEM is shown (N = 3 experiments).
(EPS)
